# The changing identity of preventive cardiology: from risk factors to risk phenotypes

**DOI:** 10.1016/j.ajpc.2026.101660

**Published:** 2026-05-07

**Authors:** Michael D. Shapiro

**Affiliations:** Department of Cardiovascular Medicine, Wake Forest University School of Medicine, Medical Center Boulevard, Winston Salem, NC 27157

For much of its modern history, preventive cardiology has been organized around the identification and treatment of individual risk factors. Elevated low-density lipoprotein (LDL)-cholesterol (LDL-C), hypertension, diabetes, smoking, obesity, suboptimal diet, and physical inactivity have formed the backbone of our clinical approach. That framework has driven major advances in risk reduction and remains central to good care.

However, preventive cardiology is entering a new phase. Increasingly, the field is being defined by the recognition of risk phenotypes. A risk phenotype refers to the particular biologic and clinical pattern through which cardiovascular risk is expressed in an individual patient. In some patients, the dominant driver is cumulative exposure to atherogenic lipoproteins. In others, it is metabolic dysfunction, visceral adiposity, insulin resistance, inflammation, chronic kidney disease, or a heart failure-prone cardiometabolic phenotype. Most often, it is some combination of these processes acting together over time. As clinicians, we need to assess how that risk is taking shape biologically and clinically in the person sitting in front of us.

This shift also reflects a deeper change in how we understand prevention itself. For many years, our model has largely been built around thresholds. Does this patient meet criteria for a statin? Should blood pressure treatment be intensified? Is the calculated risk high enough to justify a particular intervention? Those questions remain important, but they do not fully capture the task of contemporary prevention. Increasingly, we are being asked to determine what kind of risk a patient has, how far the disease process may have already progressed, and which combination of interventions is most likely to meaningfully alter trajectory.

This more mature view of prevention is especially relevant in the era of Cardiovascular-Kidney-Metabolic (CKM) syndrome paradigm. Lipids, glucose regulation, adiposity, renal dysfunction, vascular biology, and heart failure risk can no longer be understood as separate silos. They are interrelated expressions of a broader pathobiologic continuum. Atherosclerosis does not develop in isolation from insulin resistance, chronic inflammation, renal dysfunction, or long-term exposure to ApoB-containing lipoproteins. These processes interact over years, often decades, shaping both the form and the pace of disease expression.

This is one reason subclinical disease detection has become so central to contemporary preventive cardiology. Imaging can refine traditional risk assessment in powerful ways. It can distinguish predisposition from established disease and identify patients who remain asymptomatic but are clearly no longer early in the natural history of atherosclerosis. It reminds us, yet again, that the absence of symptoms is often a poor surrogate for the absence of disease.

At the same time, biomarkers are giving us a richer and more textured view of cardiovascular risk. LDL-C remains foundational, but it is not the whole story. ApoB, lipoprotein(a), inflammatory markers, markers of myocardial injury and wall stress such as hs-troponin and BNP or NT-proBNP, renal markers, and metabolic measures can reveal dimensions of risk not fully captured by the standard lipid panel or office blood pressure. Used thoughtfully, these tools can sharpen judgment and help us better understand which biology is driving risk in a given patient.

Still, preventive cardiology should resist the temptation to confuse more data with more clarity. Medicine is full of technologies that promise precision but do not always improve decision-making. Not every biomarker deserves routine use. Not every digital platform improves care. And not every claim of personalization is genuinely evidence-based. The future of the field will certainly include more sophisticated imaging, more nuanced biomarker strategies, digital tools, remote monitoring, and perhaps artificial intelligence-enabled decision support. But progress will depend on our ability to integrate them wisely, critically, and in ways that genuinely improve care.

That challenge is precisely why preventive cardiology is becoming one of the central disciplines in the future of cardiovascular medicine. Our field sits at the intersection of lipidology, hypertension, obesity medicine, diabetes, kidney disease, imaging, vascular biology, and outcomes research. Increasingly, some of the most important questions in cardiovascular care are being asked at those intersections. Which patients are truly at highest risk? Which therapies should be prioritized, combined, or intensified? Which markers of disease are actionable? Which technologies improve outcomes? Which advances genuinely simplify care?

The American Society for Preventive Cardiology has a critical role to play in helping answer those questions. Our task is not simply to keep pace with the growth of the field, but to help shape it responsibly. That means fostering education that crosses traditional boundaries. It means training clinicians to think in integrated cardiometabolic terms rather than in isolated disease silos. It means promoting research that clarifies which tools truly improve prediction, treatment selection, adherence, and outcomes. And it means ensuring that the future of prevention remains anchored in evidence rather than novelty alone.

The identity of preventive cardiology is changing. While treatment of conventional risk factors remains foundational, increasingly the field is defined by earlier recognition of disease, a more integrated understanding of cardiovascular and metabolic biology, and a growing ability to recognize meaningful differences in how risk is expressed from one patient to the next. The future of our field will continue to depend on new therapies and new technologies, but also on a more intelligent and biologically grounded understanding of how risk is expressed, how disease takes shape long before events occur, and how we can intervene with greater impact.

Michael D. Shapiro

President, American Society for Preventive Cardiology

## Declaration of competing interest

There is no conflict of interest

